# Synchronous Identification of *Entamoeba histolytica*, *Giardia intestinalis*, and *Cryptosporidium spp*. in Stool Samples Using a Multiplex PCR Assay

**Published:** 2018

**Authors:** Amir BAIRAMI, Sasan REZAEI, Mostafa REZAEIAN

**Affiliations:** 1. Dept. of Medical Parasitology and Mycology, School of Medicine, Alborz University of Medical Sciences, Karaj, Iran; 2. Dept. of Medical Parasitology and Mycology, School of Public Health, Tehran University of Medical Sciences, Tehran, Iran; 3. Center for Research of Endemic Parasites of Iran (CREPI), Tehran University of Medical Sciences, Tehran, Iran

**Keywords:** Diagnostics, *Entamoeba histolytica*, *Giardia intestinalis*, *Cryptosporidium* spp., Multiplex PCR

## Abstract

**Background:**

Diarrheal disease annually causes 760000 deaths in children, and 1700 million new cases are reported each year worldwide. Among the parasites, *Entamoeba histolytica*, *Giardia intestinalis*, and *Cryptosporidium* spp. are the most important infectious agents leading to diarrhea. Clinical presentations due to these parasites are more or less similar, and microscopy is not as much as sensitive for the detection. The aim of this study was to set up and evaluate a Multiplex PCR Assay for Synchronous Identification of *Entamoeba histolytica*, *Giardia intestinalis*, and *Cryptosporidium* spp. in Stool Samples

**Methods:**

Samples were obtained from different sources such as culture media and patient stool samples. Primer pairs were designed using primer-BLAST, and for the extraction of DNA, the QIAamp DNA stool mini kit was used. The study was conducted in Tehran, Iran and completed in 2016.

**Results:**

The current multiplex PCR assay for the detection of *E. histolytica* achieved sensitivity and specificity of 86.36% (95% CI: 65.09% to 97.09) and 95.74 % (95% CI: 85.46% to 99.48%), respectively. Sensitivity and specificity of the test for *G. intestinalis* was 90.91% (95% CI: 70.84% to 98.88%) and 95.74% (95%CI: 85.46% to 99.48%), respectively, and for the detection of *Cryptosporidium*, multiplex PCR showed a sensitivity of 90.91% (95% CI: 70.84% to 98.88%) and specificity of 95.74% (95%CI: 85.46% to 99.48%).

**Conclusion:**

Multiplex PCR in this study showed admissible sensitivity and specificity for the detection of *E. histolytica*, *G. intestinalis*, and *Cryptosporidium* spp. in fecal samples.

## Introduction

The significant second reason for death in children below five years old is the diarrheal disease, which is avoidable and treatable. Diarrhea alone amortizes 760000 children annually. Incidence of diarrheal disease is 1700 million cases each year, and in children under five years old, it is a major origin of malnutrition (1). Infectious agents are the most common causes of diarrhea consisting of viruses, bacteria, and parasites (2). Among the parasites, *E. histolytica*, *G. intestinalis*, and *Cryptosporidium* spp. are the most important (3–5).

*E. histolytica* infects 50 million individual globally and causes around 100000 deaths each year (6). *E. histolytica* and *Entamoeba dispar* which are now distinct species historically became known pathogenic and nonpathogenic strains of *E. histolytica*. In recent years’ perfect genetic, biochemical, and immunological studies, differences between *E. histolytica* and *E. dispar* are corroborated (7–9). In intestinal amoebiasis diagnosis, serological tests are of less benefit in contrast to extra intestinal entanglement (10). In less developed settings, *E. histolytica* and *E. dispar* even with impossibility to differentiation by morphology are diagnosed by microscopy (10); this is not sufficient for exact diagnosis, and ancillary approaches such as molecular detections are in demand, otherwise, microscopy will miss many infections.

*Giardia* is a prevalent gastrointestinal track parasite which causes diarrhea, abdominal pain, dyspepsia, and gas (11). In addition to viruses, *G. intestinalis* has an important role in creating diarrhea in developed countries (12). Laboratory diagnosis of *Giardia* infection is easily performed by the detection of cysts on permanent stained smears, but they are not shed continuously, and stool exam is often insufficient to represent the presence of infection (13). There are alternative detection techniques for giardia infection such as antigen detection assays such as direct fluorescent antibody (DFA) test (14) and ELISA as a practical diagnostic method (15).

*Cryptosporidium* species cause gastrointestinal infections through fecal-oral transmission by contaminated food, water, and swimming in public pools (10). *Cryptosporidium* spp. causes infections mainly in children less than 5 yr old in under developed countries, and children younger than 2 yr of age are at higher risk (16, 17). In patients with HIV/AIDS diarrhea due to cryptosporidiosis, it can become chronic and life-threatening (18).

Modified acid-fast (MAF) staining is the first line diagnostic test, but the sensitivity is only 54.8% (14). Generally, *E. histolytica, G. intestinalis,* and *Cryptosporidium* spp. showing similar presentations in the clinic are the parasites, which could cause diarrhea in the public (2). Microscopy alone is not as much as sensitive and specific for the detection of all three infections. Recently, very specific and sensitive molecular methods such as real-time PCR-based methods have been successfully introduced for of all three protozoan parasites, but for routine diagnostic purposes, this approach is too expensive (18, 19). Previously, DNA extraction was very difficult, especially from fecal samples. Recently, DNA isolation from parasites nested in the intestinal bowel has been upgraded and simplified (20).

With consideration of high sensitivity and specificity of molecular-based methods and on the other hand thinking about high cost of parasites’ identification in a routine diagnostic laboratory by molecular methods, a multiplex polymerase chain reaction was instructed in this study for the simultaneous diagnosis of *E. histolytica*, *G. intestinalis*, and *Cryptosporidium* spp. in stool specimens. These multiplex PCR assay results were compared with the microscopy as gold standard diagnosis.

## Materials and Methods

### Samples

For obtaining control DNA, samples included *E. histolytica* from an axenic culture of *E. histolytica* strain HM1, refined *G. intestinalis* cysts, and refined *Cryptosporidium* spp. oocysts.

Until 2016, with complete observation on the ethical issues and informed consent by the authors, within more than 5000 stool samples collected from different hospitals in Tehran and rural areas of Bandar-Abbas, southern Iran (Takht village, Goduo village, Gishan village, Chahestan village and etc.). Among all age groups, 22 stool samples were detected by microscopy positive for *E. histolytica,* five of coinfected with *G. intestinalis* and two were coinfected with C*ryptosporidium* spp. Moreover, 22 microscopically positive stool samples for *G. intestinalis* were used; two of them were coinfected with C*ryptosporidium* spp. Additionally, 22 stool samples were detected positive for *Cryptosporidium* spp. by microscopy on modified acid-fast prepared slides; one sample was simultaneously coinfected with both *E. histolytica* and *G. intestinalis.* Furthermore, 47 fecal samples were selected with a disaffirmed test for all three mentioned protozoan parasites by microscopy and modified acid-fast staining.

Informed consent was taken from the participants before the study and Ethics Committee of the university approved the study.

The specificity of the PCR was tested on DNAs obtained from *E. dispar, Entamoeba coli, Vibrio cholera, Escherichia coli, and Candida albicans.*

### Microscopy and modified acid-fast (MAF) staining

Microscopy for the detection of trophozoites was done on the dysenteric and watery stools directly without adding ringer for cysts after formol-ether concentration temporary iodine-stain performed on wet mounts and seen with high-power field *400 (21).

### DNA extraction

For DNA extraction, the QIAamp method was used by QIAamp DNA stool mini kit (QIAGEN, Hilden, Germany). Before using DNA stool mini kit, a pretreatment was done as follows: 0.5 gr of the specimens were washed two times with germ-free PBS and centrifuged at 14000 rpm for 5 min; freeze-thaw was performed on the stool pellet six times by putting in liquid nitrogen and 95 °C water bath repeatedly (18). DNA extraction was done by QIAamp DNA stool mini kit (QIAGEN, Hilden, Germany) according to the manufacturer’s instructions with InhibitEX tablets for elimination of PCR inhibitors from stool samples. Isolated DNA was solved in 0.2 mL of AE buffer (supplied with the QIAGEN kit) and preserved at −20 °C until examination.

### Primer designing

Primer-BLAST was used for choosing primers; Primer 3 software was used for primer designing, utilizing global alignment and BLAST algorithm to generate specific primers (NCBI/Primer-BLAST) (22).

The primers for *E. histolytica* on the cysteine protease-8 (CP8) gene (accession no. AY156066) were designed and for *G. intestinalis* on the Cathepsin L-like protease (accession no. XM_001706220) designed previously by authors (23). For *Cryptosporidium* spp., they were used from designed primers on small subunit ribosomal RNA gene (accession no. GQ259149) (20). All designed primers were ordered and bought from Cinna Gen Co (Karaj, Iran) (Table 1).

**Table 1:** Oligonucleotide primers and probes for real-time PCR assay for the simultaneous detection of *E. histolytica, G. intestinalis,* and *Cryptosporidium* spp.

***Protozoan parasite and primers name***		***Oligonucleotide sequence of primers(5′-3′)***	***GenBank accession no.***
*E. histolytica* EHCP8-As1	EHCP8-S1	ATTTGTTAAGTATTGTAAATGGG ATTGTAACCTTTCATTGTAACAT	AY156066
*G. intestinalis* GLCP6-As1	GLCP6-S1	AATCTGTTGACTTAAGGGAGTA ATTGAGTCATTATAGGGATTGT	XM_001706220
*Cryptosporidium* spp. CRY18s-As1	CRY18s-S1	TAAACGGTAGGGTATTGGCCT CAGACTTGCCCTCCAATTGATA	GQ259149

### Singleplex PCR detection setup

In a volume of 50 μL, the master mix (containing 10× PCR Buffer “20 mM (NH4)2 SO4, 75 mM Tris-HCl (pH. 8.8)”, 0.2 mM dNTP mix, 1 mM MgCl2, 1μl DNA template,1 unit/μl Taq DNA polymerase,20 pmol/μl of each primer sense & antisense, x ddH2O) was prepared for the singleplex PCR amplification reactions. Under DNase and RNase free condition after 5 min at 94 °C, 35 amplification cycles consisting of 1 min at 94 °C, 1.5 min at 55 °C, and 2 min at 72 °C for each parasite were performed separately.

For the visualizing of PCR products, 15 μL of samples was mixed with 5 μL of DNA loading buffer, and in parallel of 100bp Plus (Fermentas) Size Marker were electrophoresed. The expected band weights for *E. histolytica*, *G. intestinalis*, and *Cryptosporidium* spp. were 605, 463, and 240 bp, respectively.

### Sequencing Singleplex PCR products by designed primer pairs

For approving the accuracy of Singleplex PCR results for parasites, products of Singleplex PCR were sent to and sequenced by MWG-Biotech AG, Germany.

### Multiplex PCR detection setup

The master mix in a volume of 50 μL (containing 10× PCR Buffer “20 mM (NH4)2 SO4, 75 mMTris-HCl (pH. 8.8)”, 0.3mM dNTP mix, 3mM MgCl2, 1μl DNA template, 1.2 unit/μl Taq DNA polymerase, 20 pmol/μl of each primer sense & antisense, x ddH2O) was prepared for the Multiplex PCR amplification reaction.

Under DNase and RNase free condition after 10 min at 94 °C, 35 amplification cycles consisting of 30 seconds at 94 °C, 1.5 min at 55 °C, and 1.5 min at 72 °C, and final extension at 72 °C for 10 min followed by 1 min at 20 °C were performed. For the visualizing of PCR products, 15 μL of samples was mixed with 5 μL of DNA loading buffer and electrophoresed in parallel with Gene Ruler 100bp Plus (Fermentas) size marker.

## Results

### Evaluation of the specificity of the test

**Fig. 1:**
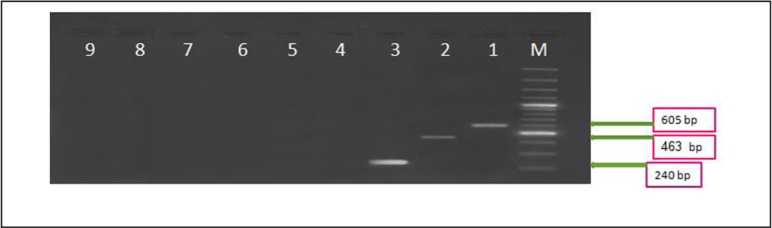
Agaros gel (1%) stained with etidiume bromide for Multiplex PCR products of primer pairs: (*E. histolytica* EHCP8-S1 EHCP8-As 1, *G. intestinalis* GLCP6-S1 GLCP6-As1, *Cryptosporidium* spp. CRY18s-S1 CRY18s-As1). M: size marker, line 1: *E. histolytica* (positive control), line 2: *G. intestinalis* (positive control), line 3: *Cryptosporidium* spp (positive control), line 4 to 8 *E. dispar* samples, line 9: negative control

Evaluation of the specificity of the multiplex PCR was done using a group of organisms: *Entamoeba dispar* (Fig. 1), *Entamoeba coli, Vibrio cholera, Escherichia coli,* and *Candida albicans*. There was no amplification in any of the determinations of these samples. Moreover, 47 DNA samples obtained from stools were tested negative for all three mentioned protozoan parasites by microscopy and modified acid-fast staining. PCR results were also positive for none of *E. histolytica*, *G. intestinalis*, and *Cryptosporidium* spp. in these samples.

### Comparison of multiplex PCR assay and singleplex PCR assays with the “gold standard” diagnosis on stool samples.

Positive fecal samples for *E. histolytica*, *G. intestinalis*, and *Cryptosporidium* spp. consisted of 22 for each and 47 negative for all three parasites by the microscopic detection as the “gold standard” diagnosis. Within 22 *E. histolytica* samples, five were also infected with *G. intestinalis*, whilst two samples were likewise positive for *Cryptosporidium* spp.; two stool samples were coinfected by *G. intestinalis* and *Cryptosporidium* spp. simultaneously, and one sample was simultaneously coinfected with all three protozoan parasites. The constructed multiplex PCR assay for detection of *E. histolytica* in comparison with the microscopy as “gold standard” test achieved a sensitivity of 86.36% (95% CI: 65.09% to 97.09%), whereas the singleplex PCR assay had a sensitivity of 90.91% (95% CI: 70.84% to 98.88%). Specificity of both multiplex and singleplex PCR assays was 95.74% (95% CI: 85.46% to 99.48%). Thus, positive predictive value (PPV) of the multiplex PCR assay for the detection of *E. histolytica* was achieved 90.48% (95% CI: 69.62% to 98.83%), and the multiplex PCR assay negative predictive value (NPV) for the detection of *E. histolytica* was 93.75% (95%CI: 82.80% to 98.69%). The accuracy of the multiplex test in the detection of *E. histolytica* was 92.75%.

Diagnostic sensitivity of the test for *G. intestinalis* with the multiplex PCR assay was 90.91% (95%CI: 70.84% to 98.88%) and 100% (95%CI: 84.56% to 100%) with singleplex PCR assay in comparison with the gold standard. Multiplex and singleplex PCR assays showed specificity of 95.74% (95%CI: 85.46% to 99.48%) and 97.87% (95%CI: 88.71% to 99.95%) in the detection of *G. intestinalis*, respectively. Thus, positive predictive value (PPV) of the multiplex PCR assay for the detection of *G. intestinalis* was achieved 90.91% (95%CI: 70.84% to 98.88%), and the multiplex PCR assay NPV was 95.74% (95%CI: 86.46% to 99.48%). The accuracy of the multiplex assay for the detection of *G. intestinalis* was 94.20%.

The multiplex PCR in analogy with the gold standard for the detection of *Cryptosporidium* showed a sensitivity of 90.91% (95%CI: 70.84% to 98.88%), whereas the singleplex PCR achieved a sensitivity of 95.45% (95%CI: 77.16% to 99.88%). Specificity of both multiplex and singleplex PCR assays for the detection of *Cryptosporidium* in stool samples was 95.74% (95%CI: 85.46% to 99.48%) and 97.87% (95%CI: 88.71% to 99.95%), respectively.

Thus, PPV of the multiplex PCR assay for the detection of *Cryptosporidium* in stool samples was achieved 90.91% (95%CI: 70.84% to 98.88%), and the multiplex PCR assay NPV was 95.74% (95%CI: 85.46% to 99.48%). The accuracy of the multiplex assay for the detection of *Cryptosporidium* in stool samples was 94.20%.

## Discussion

Diarrhea due to parasitic infections mostly occurs by *E. histolytica, G. intestinalis,* and *Cryptosporidium* spp.; infections of these agents and their broad clinical appearances make it very hard to distinguish them from other non-parasitic originators of diarrhea.

Microscopy as a usual diagnostic method applied in most laboratories for the detection of three mentioned parasites is neither sensitive nor specific.

Single PCR tests for the detection of these parasites are currently available, but multiplex PCR assay established in this study for the diagnosis of *E. histolytica*, *G. intestinalis*, and *Cryptosporidium* spp. gives an advantageous choice for the detection of these parasites.

There are three studies on multiplex real-time assay for the detection of *E. histolytica*, *G. intestinalis*, and *Cryptosporidium* spp. (18, 19, 24); the current multiplex PCR used conventional PCR protocol for the detection of parasites, but it had similar performance to the so-called reported real-time based methods.

By analyzing well-defined positive stool samples and controls, the present multiplex PCR assay for the detection of *E. histolytica*, *G. intestinalis*, and *Cryptosporidium* spp. achieved specificity of 95.74%, and for *G. intestinalis* and *Cryptosporidium* spp., multiplex PCR assay showed sensitivity of 90.91%, whereas multiplex PCR assay achieved sensitivity of 86.36% for the detection of *E. histolytica*. No significant difference was found regarding the amplification in singleplex assays in comparison with the multiplex PCR, so the multiplex PCR may be used with comparable assurance as singleplex analyzer.

Microscopy has lower sensitivity for the detection of parasites in comparison with PCR (25, 26), so to overcome this problem, the testing of six repeated stool samples was suggested. As an alternative, this multiplex PCR has the ability to cut down the need for repeated stool in routine diagnostic laboratories.

Stool contains serious PCR inhibitor materials, some of known to us and some are unknown; for reducing this risk, DNA extraction kit was used where the specific tablets were used for removing PCR inhibitors.

The primers targeted the cysteine protease-8 (CP8) gene for *E. histolytica* and Cathepsin L-as protease gene for *G. intestinalis*. For *Cryptosporidium* spp., the primers were designed on small subunit ribosomal RNA gene instead of cysteine protease genes. rRNA gene was targeted instead of cysteine protease gene for *Cryptosporidium* spp. since we were attempting to achieve equivalent annealing temperature for all the primers designed in the study.

In less developed countries, application of real-time PCR assays for the diagnostic purposes is not applicable due to its high cost in these countries, but in the developed world, multiplex real-time PCR assays and emerging DNA isolation methods along with automated approaches have excellent influences on the diagnosis of infectious agents. On the other hand, the current developed multiplex-PCR assay for the detection of *E. histolytica*, *G. intestinalis*, and *Cryptosporidium* spp. in stool samples can be employed in less developed settings because the conventional PCR thermal cyclers and reagents are now more accessible in these countries, unlike the past.

Hence, other multiplex assays could be constructed for other parasitic agents especially for those emerging parasitic infections that are more influencing the developing countries. Moreover, these new methods will benefit special groups of patients such as diabetics, patients with cancer, and immunocompromised patients as well as travelers. In addition, considering other infectious agents that cause diarrhea including viruses and bacteria, a superior panel could be completed in the differential diagnosis of these diseases in routine laboratory procedures.

## Conclusion

The presented multiplex PCR showed an acceptable sensitivity and specificity for the detection of *E. histolytica*, *G. intestinalis*, and *Cryptosporidium* spp. in fecal samples and encouraged us to believe molecular identification as a practical approach in the routine diagnostic laboratory stings for parasitic infections.
